# Combinatorial *cis*‐regulatory elements shape temporal effector expression in *Ustilago maydis*

**DOI:** 10.1111/mpp.70358

**Published:** 2026-10-04

**Authors:** Georgios Saridis, Janina Werner, Katharina Stein, Luyao Huang, Ute Meyer, Jan Muelhoefer, Namarta C. Singh, Gunther Doehlemann

**Affiliations:** ^1^ Institute for Plant Sciences University of Cologne Cologne Germany; ^2^ Cluster of Excellence on Plant Sciences (CEPLAS) University of Cologne Cologne Germany

**Keywords:** artificial promoter, *cis*‐regulatory elements, effector gene expression, *Ustilago maydis*

## Abstract

Plant‐pathogenic fungi secrete small proteins, termed effectors, to reprogramme host metabolism and suppress immune responses during infection. Although transcriptional waves of effector expression have been described in several pathosystems, the *cis*‐regulatory elements underlying their temporal coordination remain largely unknown. Here, we investigate the temporal regulation of effector genes in the biotrophic smut fungus *Ustilago maydis* , a model organism for fungal plant pathogenesis. By integrating transcriptome reanalysis with comparative promoter motif enrichment across biotrophic fungi, we identify distinct promoter motifs associated with defined infection phases. In *U. maydis* , three candidate *cis*‐regulatory elements correlate with early, proliferative and late infection stages, respectively. Positional enrichment relative to transcription start sites supports their regulatory relevance. Functional dissection of the native *pep1*, *stp1*, and *cmu1* promoters revealed strongly context‐dependent contributions of these elements and synthetic promoter assays showed that the early‐phase motif acts as an activating and the proliferative motif as a repressive element. Together, our findings support a combinatorial *cis*‐regulatory code in which activating and repressive promoter elements are integrated in a promoter‐specific manner to shape temporal effector expression during *U. maydis* infection. These elements provide a framework for engineering synthetic fungal promoters with tunable transcriptional outputs.

## Graphical abstract

We identified promoter motifs associated with infection phases in *Ustilago maydis* and analysed their positional distribution relative to transcription start sites and generated a synthetic minimal promoter that drives in‐plant gene expression.

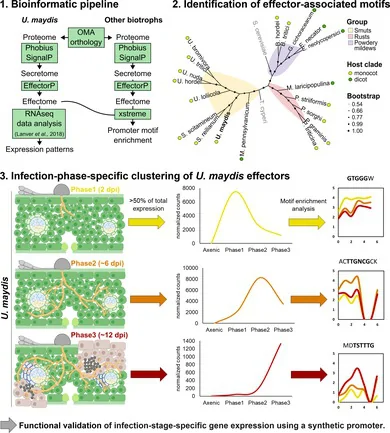

1

To effectively fulfill their infection cycle, plant‐pathogenic fungi secrete small proteins called effectors. Effectors act either in the apoplast or within host cells, where they manipulate host metabolism and suppress immune responses (Thomma et al. 2011). Because effectors promote host colonization while being subject to recognition by the host immune system, they are exposed to strong reciprocal selection pressures characteristic of the host–pathogen arms race (Ingle et al. 2006). This makes effectors rapidly evolving components of pathogen virulence and important targets for understanding host–pathogen interactions and developing disease control strategies (Fouché et al. 2018). Rapid effector diversification raises the question of whether regulatory logic may be more conserved than effector sequence itself.

Smut fungi are biotrophic pathogens of the Ustilaginales, infecting a wide range of monocot and dicot hosts, including economically important cereals, such as barley, wheat, and maize. The maize smut *Ustilago maydis* is an established model system for biotrophic fungal genetics and ranks amongst the top 10 most scientifically significant fungal pathogens (Dean et al. 2012; Matei and Doehlemann 2016). It occurs as a saprophytic, nonpathogenic haploid yeast‐like form that can be grown in artificial media, and as a biotrophic dikaryotic filamentous form, generated by the fusion of two compatible haploid cells (Kämper et al. 2006; Kruzel and Hull 2010). The infection cycle of *U. maydis* comprises (i) penetration, (ii) *in planta* proliferation and (iii) tumour formation and sporulation, the latter being the typical smut phenotype (Banuett and Herskowitz 1996; Brefort et al. 2009). These distinct developmental stages suggest that successful maize colonization requires temporally coordinated effector deployment.

Many *U. maydis* effectors have been characterized for their function and mode of action, and a number of transcriptional regulators have been identified that separate effectome activation into two waves (Djamei et al. 2023). Early effectome activation is closely linked to cell fusion and filamentation, which activate the *b* mating‐type locus genes and produce the heterodimeric bE/bW homeodomain transcription factor (Gillissen et al. 1992; Kämper et al. 1995). This master regulator of early pathogenic development in turn activates a group of effector genes as well as the zinc finger transcription factor Rbf1 and its target transcription factors Hdp2 (homeobox), Biz1 and Mzr1 (C_2_H_2_ zinc finger) (Flor‐Parra et al. 2006; Heimel et al. 2010; Zheng et al. 2008). Uncharacterized transcription factors correlated with early effectome expression contain bHLH, TEA/ATTS, HMG, CCHC and winged‐helix domains (Lanver et al. 2018). Upon *in planta* proliferation and hyphal development, the Zn_2_Cys_6_ transcription factor Zfp1 has been connected with the regulation of 112 effectors and 50 transcription factors, including Ros1 (WOPR), which successively activates the late effectome while simultaneously attenuating the wave of early effectors during sporulation and tumour formation (Cheung et al. 2021; Tollot et al. 2016). Additionally, Nlt1 (APSES), Fox1 (forkhead) as well as novel proteins containing Zn_2_Cys_6_, bHLH, C_2_H_2_, and winged‐helix domains have been associated with effector gene expression at late infection stages (Lanver et al. 2017, 2018; Zahiri et al. 2010). Despite this detailed knowledge of the transcription factor hierarchy controlling pathogenic development, the *cis*‐regulatory elements that contribute to temporal effector expression remain largely unidentified.

Here, we systematically identify promoter motifs associated with phase‐enriched effector expression in *U. maydis* using comparative genomics, transcriptome reanalysis, and functional promoter validation. By integrating evolutionary and stage‐specific analyses, we ask whether temporal effector deployment is encoded at the promoter level.

To identify putative *cis*‐regulatory elements enriched in the promoters of fungal effector genes, the genomes of different smuts, rusts and powdery mildews were analysed (Figure 1A, File S1). Protein fasta files from nine smut fungi ( *Ustilago maydis* , *U. bromivora*, *U. tritici* , *U. nuda* , *U. loliicola*, *U. hordei*, *Sporisorium reilianum*, *S. scitamineum*, *Melanopsychium pennsylvanicum*), five rust fungi (*Puccinia sorghi*, *P. striiformis* f. sp. *tritici*, *P. triticina*, *P. graminis* , *Melampsora larici‐populina*) as well as five powdery‐mildew fungi (*Blumeria hordei*, *B. graminis* f. sp. *tritici*, *Erysiphe necator*, *E. neolycopersici*, *Golovinomyces cichoracearum*) were used to identify orthologous genes among the tested species. Based on this orthology analysis, a species tree was constructed using *Saccharomyces cerevisiae* as a general outgroup, and *Testicularia cyperi* as outgroup for the smut clade (Figure 1B). In parallel, the same files were used to predict each total effectome. Transmembrane domains were predicted with Phobius and signal peptides with SignalP. Proteins with transmembrane domains and without signal peptide were filtered out, and the remaining sequences, defined as the predicted secretome, were analysed with EffectorP, leading to effectome sizes spanning from 157 in *U. loliicola* to 1266 in *P. graminis* (Table S1). The 500 bp promoter sequences of all predicted effector genes were then used to identify enriched motifs with XSTREME from the MEME Suite, and motifs passing the thresholds (*p* < 0.05 and enrichment sites > 10%) were compared using STAMP. The smut *M. pennsylvanicum* and the powdery mildews *E. necator*, *G. cichoracearum* and *E. neolycopersici* showed no significant motif enrichment. Notably, one motif with consensus sequence GTGGG was specifically enriched in the effectome promoters of all tested smuts but not in rusts and powdery mildews (Figure S1). Two rust‐specific enrichments were identified with consensus sequences KCGA and TGCK, while further consensus motifs derived from either smuts and powdery mildews (ATGAA), powdery mildews and rusts (CGTG), or smuts and rusts (CWGTR and CGGCS) (Figure S1).

**FIGURE 1:**
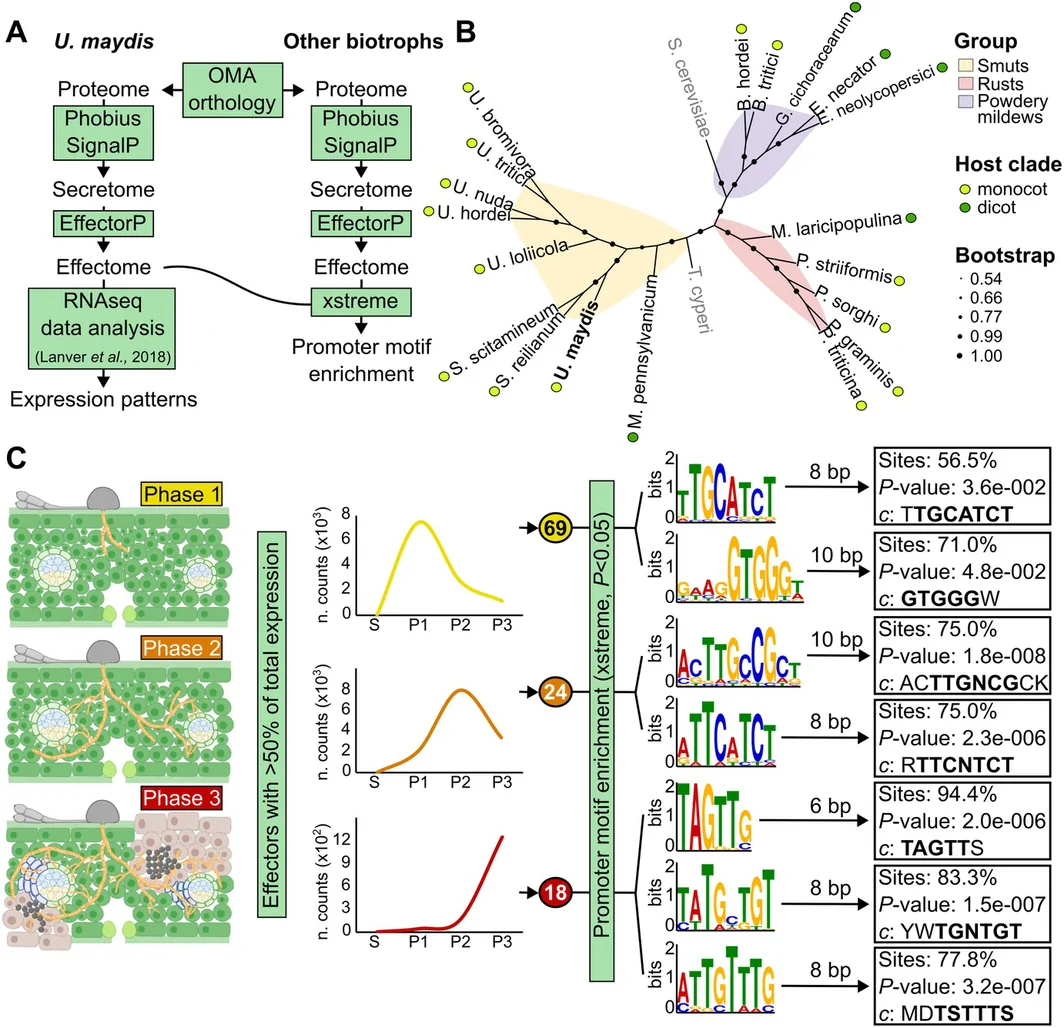
Identification of putative phase‐associated motifs in *Ustilago maydis* effectome promoters. (A) Workflow of the bioinformatic analysis for the prediction of putative effectomes in the tested species used for the promoter enrichment analysis. (B) Phylogenetic tree illustrating the evolution of the species used in this study. (C) Selection of phase‐enriched effector genes in *U. maydis* and promoter motif enrichment. Motifs with > 55% enriched sites and an enrichment *p* < 0.05 are shown and were selected for subsequent analyses. Bold letters in the motif consensus (labelled as *c*) indicate a putative core sequence. n. counts: normalized counts in counts per million.

To dissect phase‐specific effector regulation in *U. maydis* , we re‐analysed RNA‐seq data from Lanver et al. (2018) and grouped infection into three biologically defined stages: (i) Phase 1 (0.5–2 days post‐inoculation [dpi]; penetration and early colonization), (ii) Phase 2 (4–6 dpi; hyphal proliferation), and (iii) Phase 3 (8–12 dpi; tumour formation and sporulation) (Figure 1C, Table S2). Effector genes were assigned to a phase if more than 50% of their cumulative expression occurred within that interval, which yielded 69 Phase 1‐, 24 Phase 2‐ and 18 Phase 3‐enriched effectors (Figure 1C, Table S3), consistent with temporally structured transcriptional waves during infection. To test whether the phase classification depended on the chosen cut‐off, we re‐derived effector assignments across thresholds from 0.3 to 0.7 (Table S7). Phase 1 remained the largest effector set at every threshold (164, 115, 69, 40 and 27 effectors at thresholds of 0.3–0.7, respectively), and no effector was assigned to a different phase at any threshold: the Phase 1 sets were strictly nested, with less stringent cut‐offs recruiting additional effectors and more stringent cut‐offs retaining progressively phase‐restricted subsets. The threshold therefore determines only how phase‐restricted an effector must be to be included, and not the phase to which it is attributed, indicating that the temporal structure of the effectome is not an artefact of the classification criterion.

Promoter regions (500 bp upstream) of phase‐enriched effectors were subjected to motif enrichment analysis using XSTREME. Seven significantly enriched motifs (*p* < 0.05; enrichment sites > 55%) were identified across the three infection phases. Two motifs were associated with Phase 1 (TGCATCT [P1‐1] and GTGGG [P1‐2]), two with Phase 2 (TTGNCG [P2‐1] and TTCNTCT [P2‐2]), and three with Phase 3 (TAGTT [P3‐1], TGNTGT [P3‐2], and TSTTTS [P3‐3]) (Figure 1C). Among these, GTGGG (Phase 1), TTGNCG (Phase 2), and TSTTTS (Phase 3) displayed the strongest phase‐restricted enrichment patterns and were prioritised for further analysis.

To assess motif conservation and specificity, we calculated the enrichment of every motif in effectome versus non‐effectome (background) promoters of each tested species. For the motif TGCATCT [P1‐1], 6 out of the 9 tested smuts showed enrichment in the effectome, with *U. maydis* demonstrating the highest enrichment, while for *S. scitamineum*, *S. reilianum* and *M. pennsylvanicum*, the enrichment was towards the background (Figure 2A). No significant interclade differences were observed. For the other motif of Phase 1, GTGGG [P1‐2], an interclade difference was present, with all smut motifs clustering closely and demonstrating a strong preference of this motif towards smut effectome promoters (Figure 2A). Motif TTGNCG [P2‐1] revealed an overall negative enrichment (towards the background) for all fungal clades; however, individual species ( *U. maydis* , *U. bromivora*, *S. scitamineum*, *P*. *sorghi*, *P. graminis* , *B. hordei*, *G. cichoracearum*) showed effectome enrichment of the P2‐1 motif. Motif TTCNTCT [P2‐2] had a powdery mildew‐specific enrichment, with all its tested species demonstrating effectome enrichment, while from smuts, only 4 out of 9 ( *U. maydis* , *U. loliicola*, *S. reilianum* and *M. pennsylvanicum*) showed a similar pattern. *U. maydis* had again the strongest enrichment. Motifs TAGTT [P3‐1] and TGNTGT [P3‐2] demonstrated very similar patterns, with a preference towards smut effectomes, and the highest enrichment in *U. maydis* , the only exceptions being *U. hordei* for motif P3‐1 and *U. tritici* for P3‐2 (Figure 2A). Finally, motif TSTTTS [P3‐3] showed an overall enrichment towards the background for smuts and rusts and a slight enrichment towards the effectome of powdery mildews, with *U. maydis* , *S. reilianum* and *U. tritici* being the only smuts with an effectome enrichment (Figure 2A).

**FIGURE 2:**
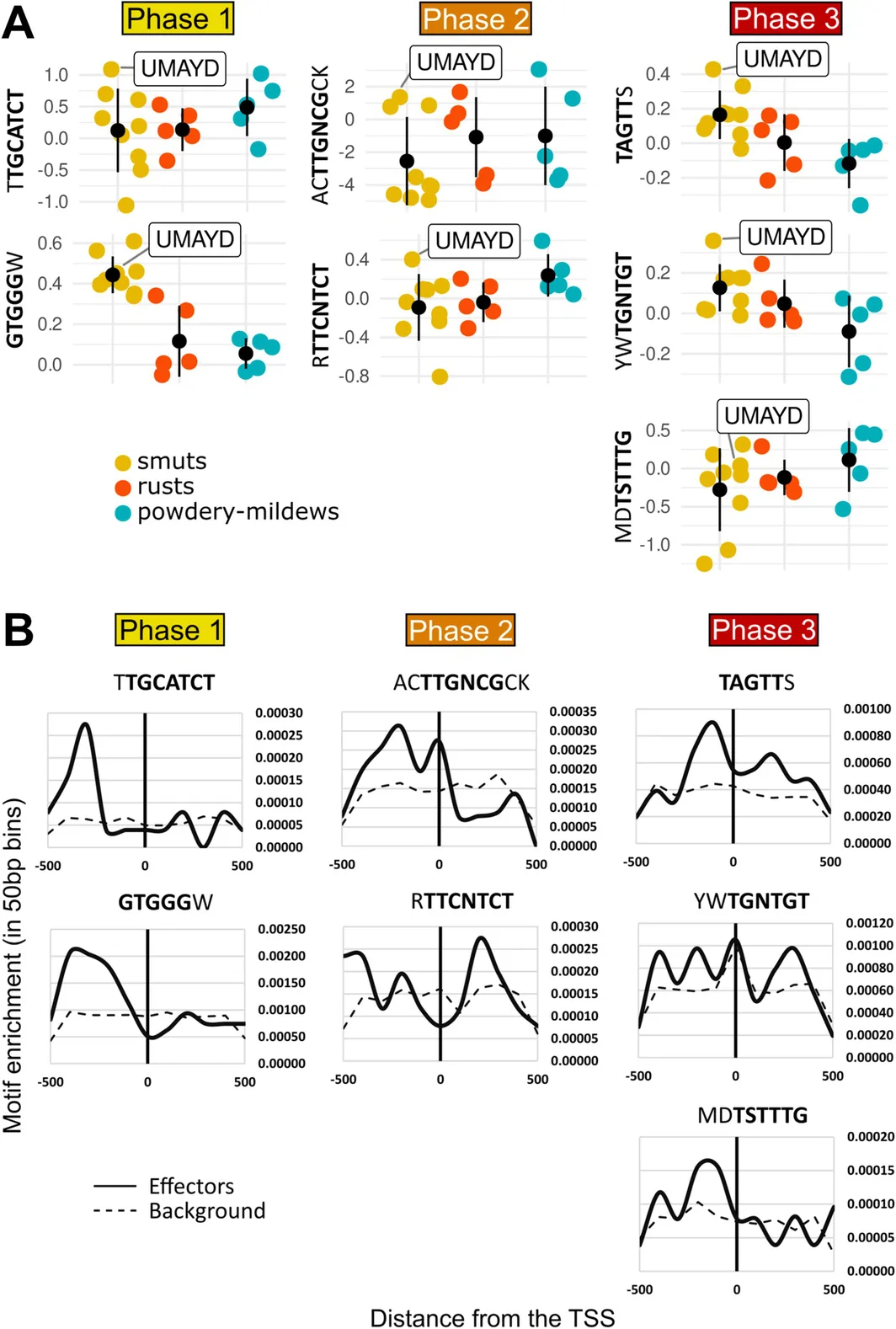
Specificity of motif enrichment within and among fungal clades and motif presence around the transcription start site (TSS) in *Ustilago maydis* . (A) Coloured dots represent the enrichment of each motif in the effectome of a species versus the non‐effectome. Black dots and error bars represent the mean ± SD within each fungal clade. Bold letters of the motifs indicate the putative core sequence. (B) Motif occurrence was calculated in all effector and non‐effector (background) gene promoters in 50‐bp bins across a 1‐kb region centred on the TSS. The average enrichment for each bin was then calculated and the enrichment curve was plotted. Bold letters of the motifs indicate the putative core sequence. Solid line: effectome, dashed line: non‐effectome.

Next, the occurence of each motif in a 1 kb region spanning the transcription start site (TSS) was calculated for both the effectome and the non‐effectome background, and their frequencies were plotted (Figure 2B). Several motifs showed non‐random positional enrichment upstream of the TSS. GTGGG [P1‐2] displayed a broad enrichment between 150 and 450 bp upstream of the TSS, consistent with promoter‐proximal regulatory regions. TTGNCG [P2‐1] showed enrichment across the 0–450 bp upstream region, whereas TSTTTS [P3‐3] exhibited discrete upstream peaks. Some motifs also displayed downstream enrichment. Overall, the positional bias of multiple motifs supports their potential regulatory function. We further tested whether known transcription factor binding sites correlate with our motifs by subjecting their consensus core sequences to TomTom (MEME Suite) analysis, against the JASPAR Fungal database. The P1‐1 motif could be similar to an APSES‐type binding site (*p* = 0.001) and the P1‐2 motif showed similarity to C2H2 zinc‐finger transcription factor binding motifs (*p* < 0.001), while the P2‐1 motif might resemble another C_2_H_2_ binding site (*p* = 0.002) and the P2‐2 motif showed similarity to a heat shock factor binding motif (*p* = 0.002). Finally, the P3‐1 motif showed similarity to binding motifs of a homeodomain (*p* = 0.015), the P3‐2 motif of a bZIP (*p* = 0.029) and the P3‐3 motif of a forkhead/winged helix (*p* = 0.008) transcription factor (Table S4).

To investigate the phase‐specificity of the identified motifs, we correlated effectome gene expression with motif occurrence in their promoters. We first tested whether motif copy number was associated with the maximum expression potential of individual effector genes. GTGGG [P1‐2] showed a positive association, and promoters containing one or two GTGGG copies exhibited elevated early‐stage expression compared to promoters lacking the motif (Figure S2). To further dissect the possible influence of each motif on phase‐specific expression, *U. maydis* effectors were then split into groups based on the number of occurrences of each motif in their 500 bp promoters, ranging between 0 and 6, and the average expression of each group was calculated separately for Phases 1–3 (Figure 3A). Motif TGCATCT [P1‐1] showed highest expression with up to two copies but no phase specificity. TTGNCG [P2‐1] enabled expression with 0–3 or 6 copies, with a strong Phase 2 preference at 1–3 copies. TTCNTCT [P2‐2] resembled [P1‐1], allowing expression only up to two copies without phase specificity. TAGTT [P3‐1] showed a similar pattern, with slight Phase 3 preference. TGNTGT [P3‐2] exhibited higher expression at 1, 3 or 4 copies, with weak Phase 3 specificity at 1 or 4 copies. Finally, the motif TSTTTS [P3‐3] had the clearest specificity towards expression at Phase 3, especially when 3 or 4 copies were present in the promoters (Figure 3A).

**FIGURE 3:**
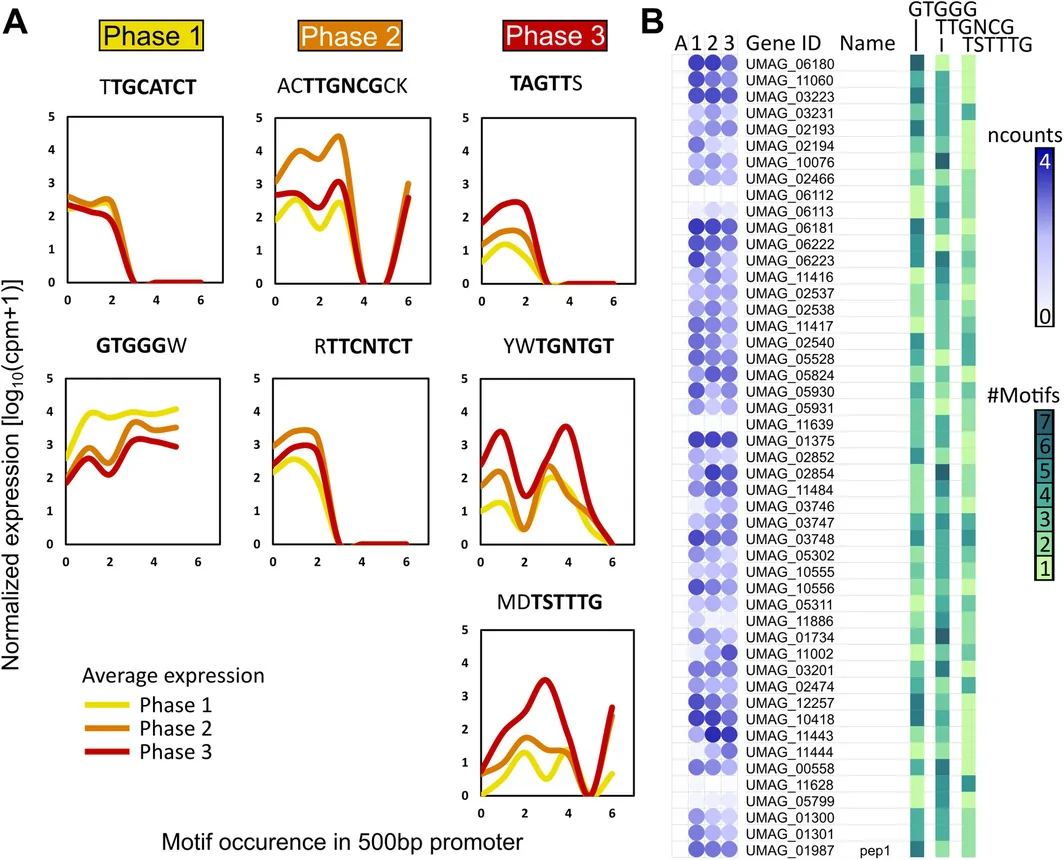
Association of motif occurrence with phase‐enriched effector expression. (A) Relationship between promoter motif copy number and mean effector expression across the three infection phases. The average expression value for each phase was calculated for all the genes with the same amount of each motif in their 500 bp promoter. Bold letters of the motifs indicate the putative core sequence. (B) Selected genes which contain all three of the candidate phase‐specific motifs. Heatmap (circles) depicts normalized counts (ncounts, in log_10_(cpm + 1)). The numbers of each motif are shown in the right panel.

The three prioritized motifs, namely GTGGG [Phase 1; P1‐2], TTGNCG [Phase 2; P2‐1] and TSTTTS [Phase 3; P3‐3], were used to identify the most fitting effector candidates for follow‐up characterization. 49 effectors containing all three motifs in their 500 bp promoters were selected and their expression was determined using the re‐analysed RNA‐seq dataset from Lanver et al. (2018) (Figure 3B).

To experimentally test whether enriched motifs directly influence effector gene expression, we performed promoter mutagenesis using the *pep1*, *stp1*, and *cmu1* promoters to drive GFP expression at the *ip* locus. For *pep1* six GTGGG motifs (positions −56, −160, −240, −300, −394, −584), two TTGNCG motifs (−671, −839), and two TSTTTS motifs (−720 and −885) were mutated. For *stp1*, three GTGGG motifs (−209, −240, −319) and six TSTTTS motifs (−44, −400, −408, −576, −653, −854) were mutated. For *cmu1*, six GTGGG motifs (−274, −322, −333, −353, −579, −890), one TTGNCG motif (−870), and one TSTTTS motif (−779) were mutated (Figure 4A). All mutations introduced the least probable nucleotide at the respective position, according to the confusion matrix of the identified motif (Table S5). Reporter expression was analysed at 3, 6 and 10 dpi, corresponding approximately to the three infection phases defined above.

**FIGURE 4:**
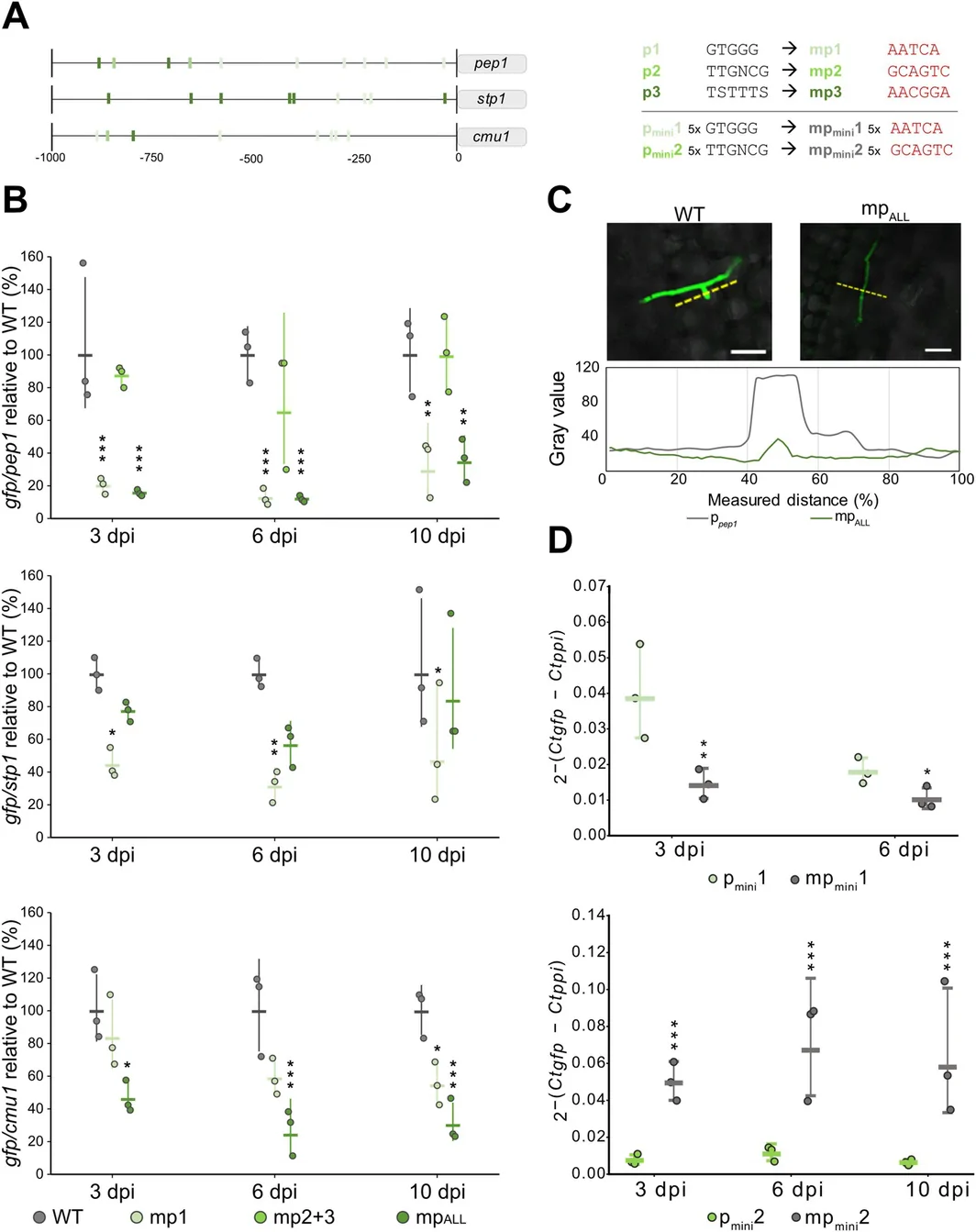
Context‐dependent activities of phase‐associated *cis*‐regulatory elements in native and synthetic promoters. (A) Schematic representations show the positions of P1‐, P2‐ and P3‐associated motifs within the 1‐kb promoter fragments of *pep1*, *stp1* and *cmu1* used to drive *gfp* expression. The corresponding motif consensus sequences and the sequences used for motif disruption (labelled in red) are indicated above the schematics. Reporter expression was analysed for the wild‐type promoters (WT), promoters carrying mutations in the indicated motifs (mp1 or mp2 + 3), and promoters in which all selected motifs were mutated (mp_ALL_). (B) Expression driven by native *pep1*, *stp1* and *cmu1* promoters carrying mutations in the indicated phase‐associated *cis*‐regulatory elements. Reporter (*gfp*) expression was quantified relative to expression of the housekeeping gene *ppi* and normalized to the respective wild‐type promoter at each time point (WT = 100%). (C) Confocal microscopy images of maize seedlings infected with p_*pep1*_‐GFP or mp_ALL_‐GFP strains at 2 dpi. The dashed yellow line indicates the region used for GFP intensity measurements. GFP intensity profiles were quantified along the dashed yellow line using ImageJ. (D) Functional analysis of isolated Phase 1 (p_mini_1)‐ and Phase 2 (p_mini_2)‐associated motifs in synthetic promoters. Five tandem copies of the intact Phase 1 (p1) or Phase 2 (p2) motif, or the corresponding mutated motifs (mp1 and mp2), were introduced into a synthetic promoter driving *gfp* at the *ip* locus of *Ustilago maydis* . Reporter expression was quantified relative to fungal *ppi* as 2^−(Ct*gfp*−Ct*ppi*)^. The Phase 1 motif increased promoter activity relative to mp_mini_1, whereas the Phase 2 motif strongly restricted expression and mutation of the motif (mp_mini_2) resulted in derepression. Dots represent three independent biological replicates; technical reverse transcription‐quantitative PCR replicates were averaged before analysis, horizontal lines indicate geometric means, and error bars represent ±1 SD in Δ*C*_t_ space after back‐transformation. Statistical analyses were performed on Δ*C*_t_ values. For native promoter analyses in (A), two‐way ANOVA with promoter genotype and time as factors was followed by Dunnett‐adjusted comparisons of each mutant with WT within each time point. For synthetic promoter analyses in (C), two‐way ANOVA with motif variant and time as factors was followed by prespecified intact‐versus‐mutated comparisons within each time point, with *p* values adjusted using the Holm method. *p < 0.05, **p < 0.01, ***p < 0.001; ns, not significant. Exact *p‐*values for all statistical tests and multiple‐comparison analyses are provided in Table S8.

For *pep1*, three promoter variants were compared with the wild‐type promoter: mp1 (GTGGG motifs mutated); mp2 + 3 (TTGNCG and TSTTTS motifs mutated); and mp_ALL_ (all selected motifs mutated). For *stp1* and *cmu1*, mp1 and mp_ALL_ promoter variants were generated. Quantification of *gfp* expression revealed promoter‐specific contributions of the selected *cis*‐regulatory elements to expression *in planta* (Figure 4B). In the *pep1* promoter, mutation of the Phase 1‐associated GTGGG elements strongly reduced reporter activity at all three time points, whereas combined mutation of the Phase 2‐ and Phase 3‐associated elements had little effect. The mp_ALL_ promoter showed a similarly strong reduction, identifying GTGGG as the dominant positive regulatory input under the tested conditions. Consistently, confocal imaging at 2 dpi showed reduced GFP signal in the mp_ALL_ strain relative to the wild‐type *pep1* promoter (Figure 4C). Phase 1‐associated elements also contributed to *stp1* promoter activity across all three time points, with the strongest effect observed at 6 dpi (Figure 4B). In *cmu1*, mutation of the Phase 1‐associated motifs had little effect at 3 and 6 dpi but caused a modest reduction at 10 dpi, whereas disruption of all selected motifs significantly reduced promoter activity at all three time points (Figure 4B). Together, these results show that the contribution of an individual motif class depends strongly on its native promoter context.

To test whether the Phase 1‐ and Phase 2‐associated motifs can exert regulatory activity outside their native promoter context, we generated synthetic minimal promoters containing five tandem copies of the intact or mutated motifs (p_mini_1 and p_mini_2) driving *gfp* expression (Table S6). Five GTGGG copies enhanced reporter expression relative to the corresponding mutated Phase 1 motif (AATCA), demonstrating autonomous positive regulatory activity (Figure 4D). Conversely, five copies of the Phase 2‐associated motif strongly restricted reporter expression, whereas motif disruption (GCAGTC) resulted in pronounced derepression, identifying this element as a sequence‐dependent repressive module. Neither synthetic system reproduced strict phase specificity (Figure 4D). Thus, the Phase 1‐ and Phase 2‐associated motifs can function autonomously as opposing regulatory modules, whereas their effects in native promoters depend on the surrounding *cis*‐ and *trans*‐regulatory context.

Biotrophic infection requires not only an appropriate effector repertoire but also the precise temporal coordination of its expression. The distinct developmental transitions of the *U. maydis* infection cycle impose different physiological and host‐interaction demands, including immune suppression, metabolic manipulation, and tissue reprogramming, making it plausible that the pathogen deploys its effectors in a temporally structured manner, consistent with previous findings that effector gene expression occurs in an organ‐ and cell type‐specific manner (Matei et al. 2018; Skibbe et al. 2010). While the hierarchical transcription factor cascades orchestrating pathogenic development in *U. maydis* have been extensively characterised (Djamei et al. 2023; Heimel et al. 2010; Lanver et al. 2017, 2018; Romeis et al. 1997), the *cis*‐regulatory logic underlying temporally coordinated effector expression at the promoter level has remained largely unresolved.

Here, we identified infection‐phase‐associated promoter motifs, analysed their positional distribution relative to transcription start sites, and provided functional evidence that distinct motifs can exert activating or repressive regulatory activity. Re‐analysis of available RNA‐seq data (Lanver et al. 2018) revealed clearly defined phase‐enriched effector clusters, consistent with previous descriptions of transcriptional waves during infection (Djamei et al. 2023; Lanver et al. 2017, 2018), and our identification of promoter motifs enriched within these clusters suggests that temporal effector deployment is, at least in part, encoded at the *cis*‐regulatory level. Whether these phase‐enriched motifs represent direct binding sites of known regulators or define additional regulatory layers remains to be determined. Comparative motif analysis across multiple biotrophic fungi further revealed that the GTGGG motif is consistently enriched in smut effectome promoters but not in rusts or powdery mildews, suggesting lineage‐specific differences in effector promoter architecture.

Certain motifs also displayed enrichment downstream of annotated TSS. Interpretation of these positions is constrained because no experimental genome‐wide TSS map exists for *U. maydis* and untranslated region (UTR) coordinates are not defined. Consequently, sequences that are functionally promoter‐proximal or located within genuine 5′ UTRs may be assigned nominally downstream positions. Experimental TSS mapping at single‐nucleotide resolution, for example by CAGE or TSS‐seq across infection stages, will be required to resolve the precise position of these motifs relative to transcription initiation.

Among the identified motifs, GTGGG [P1‐2] showed the strongest association with Phase 1 expression and its mutation identified it as the dominant positive regulatory input within the *pep1* promoter under the tested conditions, an activity that was confirmed by tandem GTGGG elements in the synthetic promoter assay. Importantly, GTGGG activity was not restricted to Phase 1, indicating that its association with early effector expression does not translate into an autonomous temporal switch.

Pep1 is a key virulence factor required for successful biotrophic colonization (Doehlemann et al. 2009), making regulation of its expression particularly relevant for host compatibility. Motif similarity analysis revealed similarity of GTGGG to C_2_H_2_‐type transcription factor binding motifs, which is compatible with the extensive transcriptional network controlling early pathogenic development, including bE/bW and downstream regulators such as Rbf1, Biz1, Hdp2, and Mzr1 (Flor‐Parra et al. 2006; Gillissen et al. 1992; Heimel et al. 2010; Kämper et al. 1995; Zheng et al. 2008). Later infection stages involve regulators such as Zfp1 and Ros1 (Cheung et al. 2021; Tollot et al. 2016). However, motif similarity alone cannot establish direct binding, and identification of the cognate transcription factor will be required to position GTGGG mechanistically within this network. The contribution of the same motif class differed markedly among native promoters. Whereas both *pep1* and *stp1* depended strongly on intact Phase 1‐associated elements, *cmu1* was comparatively robust to their mutation alone and showed a pronounced reduction only when all selected motifs were disrupted. Notably, *pep1* and *stp1* display nearly identical temporal expression profiles (Lanver et al. 2018) and encode components of the same Stp complex implicated in effector translocation, raising the possibility that shared P1‐associated regulatory inputs contribute to the coordinated expression of functionally linked complex components during early biotrophic development (Ludwig et al. 2021). Context dependence was also evident for the Phase 2 associated element, which acted as a strong repressive module in the synthetic promoter but whose combined mutation with Phase 3 elements had little detectable effect in native *pep1*.

Together, these findings support a model in which temporal effector expression emerges from the integration of activating and repressive *cis*‐regulatory inputs with promoter‐specific architecture and stage‐dependent *trans*‐acting factors, rather than from individual motifs functioning as autonomous temporal switches. Several limitations remain. The identified motifs are short and may therefore occur by chance, although their enrichment, positional bias, association with phase‐enriched expression, and experimental perturbation collectively support their regulatory relevance. In addition, Phases 2 and 3 elements were mutated together in *pep1*; the Phase 3‐associated element has not yet been tested independently, and the corresponding *trans*‐acting factors remain unknown.

Beyond their mechanistic implications, the identified elements provide potential building blocks for synthetic manipulation of fungal gene expression. Future studies should define the autonomous regulatory activity of the Phase 3‐associated element, resolve the individual contributions of Phase 2 and Phase 3 motifs in native promoters, determine how motif combination, spacing, and copy number influence regulatory output, and identify the corresponding *trans*‐acting factors. These approaches should reveal how promoter architecture integrates activating and repressive inputs into temporally coordinated effector expression and may ultimately enable the rational design of tunable fungal promoters.

## Author Contributions

**Georgios Saridis:** conceptualization, investigation, writing – original draft, methodology, software. **Janina Werner:** conceptualization, investigation, writing – original draft, supervision. **Katharina Stein:** investigation. **Luyao Huang:** investigation. **Ute Meyer:** investigation. **Jan Muelhoefer:** investigation. **Namarta C. Singh:** investigation. **Gunther Doehlemann:** conceptualization, funding acquisition, writing – review and editing, supervision, project administration.

## Funding

This project has received funding from the European Research Council (ERC) under the European Union's Horizon 2020 research and innovation programm (grant agreement no. 771035), as well as from the Cluster of Excellence on Plant Sciences (CEPLAS) funded under Germany's Excellence Strategy—EXC 2048/1—project ID: 390686111.

## Conflicts of Interest

The authors declare no conflicts of interest.

## Supporting information


**Figure S1:** Comparison of motifs enriched in the effectome promoters of biotrophic fungi. The motifs were identified as enriched in the effectome promoters of the tested smuts, rusts and powdery‐mildew species, with *p* < 0.05 and enrichment sites > 10%. Some species had more than one motif passing the set thresholds, which is reflected as a number at the motif code. Motifs were compared with STAMP and the UPGMA tree was generated with the same tool. The consensus core sequences of the groups generated after cutting the tree at the specified location (red line) were manually curated.

**Figure S2:** Correlation between motif presence and gene expression potential. Promoter motif counts were correlated with the maximum gene expression levels using Spearman's rank correlation coefficient. Gene expression values were log_2_‐transformed (log_2_(*x* + 1)). Scatter plots were visualized with slight horizontal jitter to reduce overplotting. Linear regression lines are shown for visualization purposes only. Motif cores were highlighted in bold within the full consensus sequence. The *R* statistic and *p*‐value of the correlation is depicted.

**File S1:** Experimental procedures.

**Table S1:** Details of the genomes of biotrophic fungi used in this study.

**Table S2:** Re‐analysed effector expression data from Lanver et al. (2018).

**Table S3:** Expression of *Ustilago maydis* effectome.

**Table S4:** Estimation of the most probable transcription factor group associated with the phase‐specific motifs.

**Table S5:** Confusion matrix.

**Table S6:** Sequence of artificial promoters.

**Table S7:** Assignment‐threshold sensitivity. Stability versus the 50% threshold assignment.

**Table S8:** Statistical analysis of native and synthetic promoter reporter assays shown in Figure 4.

## Data Availability

All source data are provided in this paper and its Supporting Information. All microbial strains are available on request from the corresponding author.
