# Supplementary material for: Combinatorial cis‐regulatory elements shape temporal effector expression in Ustilago maydis
Source: Mol Plant Pathol. 2026 Oct 4;27(10):e70358. doi: 10.1111/mpp.70358 (PMC13635618; doi:10.1111/mpp.70358)
Supplement: Supplementary file 1 [file MPP-27-e70358-s006.pdf]

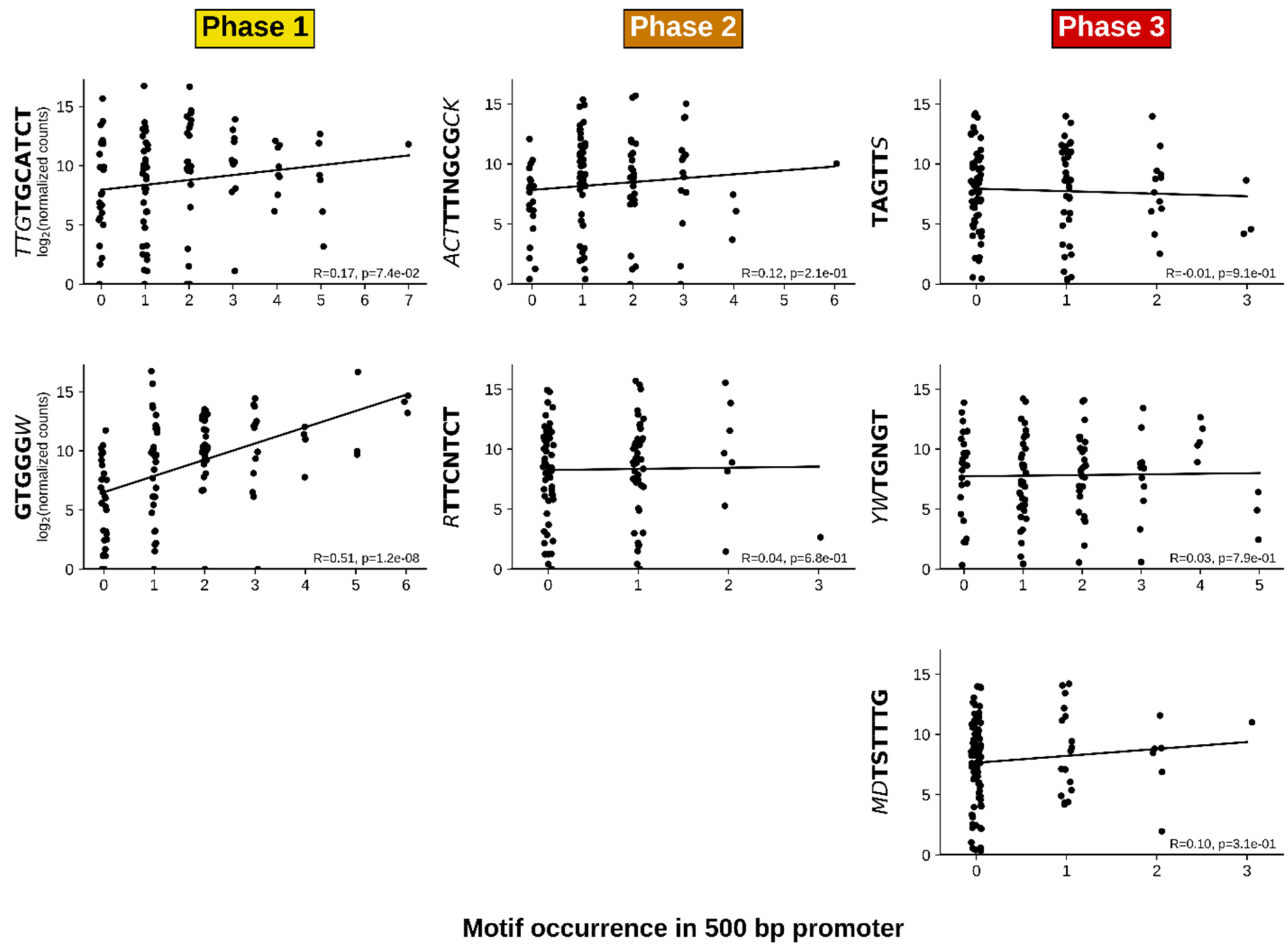

Figure S2: Correlation between motif presence and gene expression potential. Promoter motif counts were correlated with the maximum gene expression levels using Spearman's rank correlation coefficient. Gene expression values were log$_2$-transformed (log$_2$(x+1)). Scatter plots were visualized with slight horizontal jitter to reduce overplotting. Linear regression lines are shown for visualization purposes only. Motif cores were highlighted in bold within the full consensus sequence. The *R* statistic and *P*-value (p) of the correlation is depicted.
