# Supplementary material for: Combinatorial cis‐regulatory elements shape temporal effector expression in Ustilago maydis
Source: Mol Plant Pathol. 2026 Oct 4;27(10):e70358. doi: 10.1111/mpp.70358 (PMC13635618; doi:10.1111/mpp.70358)
Supplement: Supplementary file 2 [file MPP-27-e70358-s007.pdf]

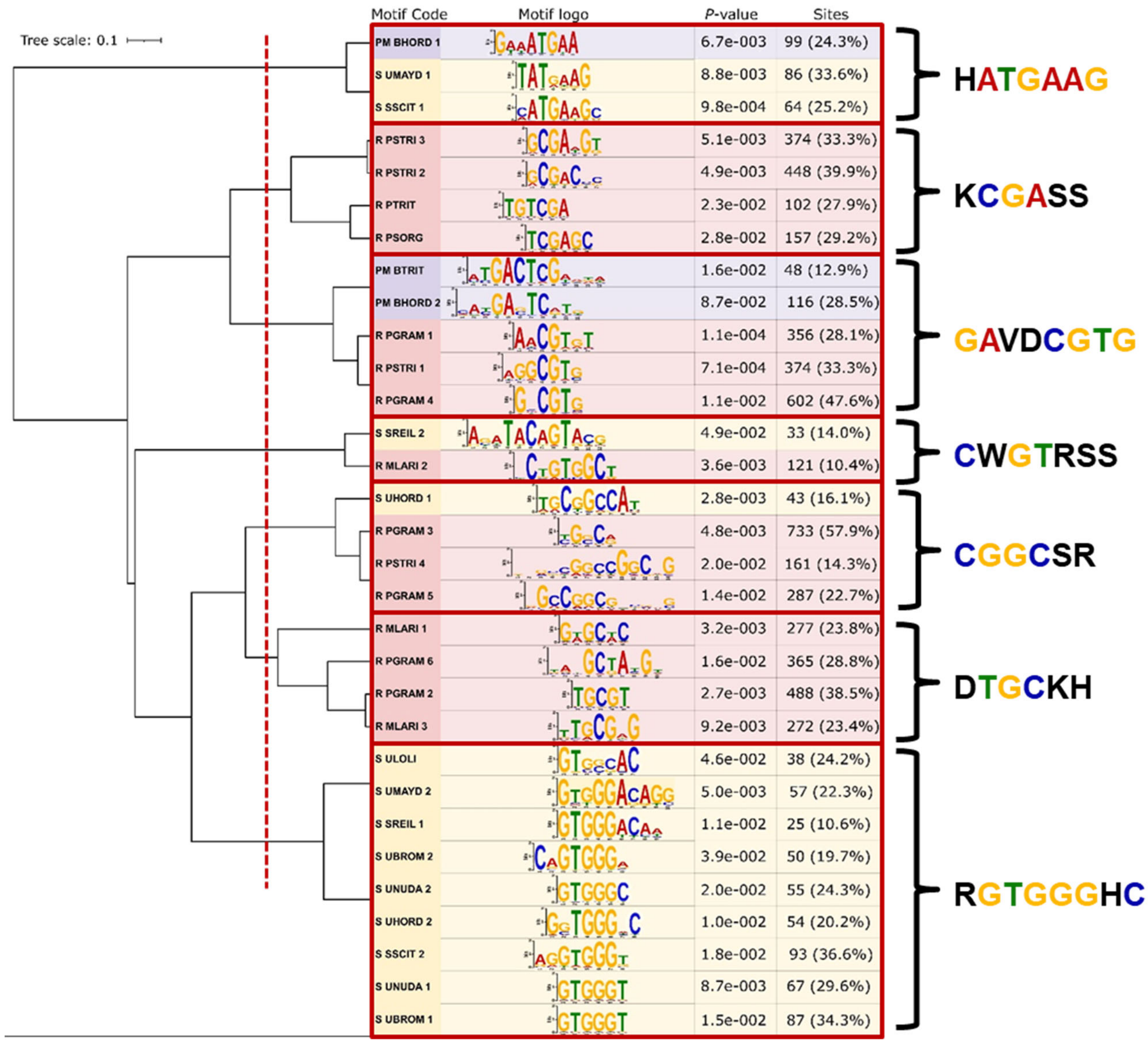

Figure S1: Comparison of motifs enriched in the effectome promoters of biotrophic fungi. The motifs were identified as enriched in the effectome promoters of the tested smuts, rusts and powdery-mildew species, with *p*-value < 0.05 and enrichment sites > 10%. Some species had more than one motif passing the set thresholds, which is reflected as a number at the motif code. Motifs were compared with STAMP and the UPGMA tree was generated with the same tool. The consensus core sequences of the groups generated after cutting the tree at the specified location (red line) were manually curated.
