# Supplementary material for: Combinatorial cis‐regulatory elements shape temporal effector expression in Ustilago maydis
Source: Mol Plant Pathol. 2026 Oct 4;27(10):e70358. doi: 10.1111/mpp.70358 (PMC13635618; doi:10.1111/mpp.70358)
Supplement: Supplementary file 3 [file MPP-27-e70358-s005.docx]

# Experimental Procedures

**Prediction of total effectomes**

The genomes, including chromosome and protein fasta files as well as annotation (GFF3 or GTF) files of 9 smuts: *Ustilago maydis* (GCA_000328475.2), *U. bromivora* (GCA_900080155.1), *U. tritici* (GCA_022963115.1), *U. nuda* (GCA_022963125.1), *U. loliicola* (GCA_022963135.1), *U. hordei* (GCA_900519145.1), *Sporisorium reilianum* (GCA_000230245.1), *S. scitamineum* (GCA_900002365.1), and *Melanopsychium pennsylvanicum* (GCA_900208305.1); 5 rusts: *Puccinia sorghi* (GCA_001263375.1), *P. striiformis* f. sp. tritici (GCA_000223505.1), *P. triticina* (GCA_000151525.2), *P. graminis* (GCA_000149925.1) and *Melampsora larici-populina* (GCA_000204055.1); and 5 powdery-mildews: *Blumeria hordei* (GCA_900237765.1), *B. graminis* f. sp. tritici (GCA_000418435.1), *Erysiphe necator* (GCA_000798715.1), *E. neolycopersici* (GCA_003610855.1) and *Golovinomyces cichoracearum* (GCA_003611235.1) were used in this study. For the prediction of the effectome, the protein fasta files were firstly subjected to an analysis with Phobius for the filtering of proteins without transmembrane domains (Käll *et al*., 2004). Thereafter, SignalP 6.0 was used for the filtering of the proteins which contain a signaling peptide, using the slow mode for eukarya (Teufel *et al*., 2022). Those proteins at this point, were considered as total secretome. For the determination of the total effectome, the total secretome of each species was used in an analysis with EffectorP 3.0 (Sperschneider & Dodds, 2022).

**Determination of orthologous genes**

For the determination of gene orthologs among the tested species, the Orthologous Matrix standalone toolkit (OMA 2.5.0) was used (Altenhoff *et al*., 2019; Train *et al*., 2017). For this, the protein fasta files from all abovementioned genomes were used. In addition, to have outgroups for fungi and smuts, the protein fasta files for *Saccharomyces cerevisiae* (GCA_000146045.2), named yeast, and *Testicularia cyperi* (GCA_003144125.1) were used, respectively. The following parameters were modified in the parameters.drw file: OutgroupSpecies:=[‘yeast’], UseOnlyOneSplicingVariant:=false.

**Phylogenetic tree construction**

The estimated species tree generated by the OMA standalone toolkit was used for the generation of the phylogenetic tree in Figure 1B. The inferred species tree in newick format was introduced to iTOL for visualization using the unrooted mode.

**RNAseq data analysis**

Paired-end RNAseq reads from Lanver *et al*. (2018) were quality trimmed using Trimmomatic, selecting for per-base quality score in the start and end of the reads ≥ 20. Subsequently, reads were mapped to the *U. maydis* genome (GCA_000328475.2) with HISAT2 in stranded mode (Kim *et al*., 2019). Thereafter, successfully mapped reads were sorted with SAMtools and counted with HTseq-count using intersection‑nonempty mode for stranded reads (Anders *et al*., 2015; Danecek *et al*., 2021). Reads deriving from the samples of 0.5, 1 and 2 dpi were added together to generate the Phase 1 timepoint; reads from 4 and 6 dpi, gave the Phase 2 timepoint; and subsequently, reads from 8 and 12 dpi were merged to create the Phase 3 timepoint. The differential gene expression analysis was performed with edgeR (Robinson *et al*., 2009), using a quasi-likelihood negative binomial generalized log‑linear model to fit the count data. Normalized counts were calculated as counts per million mapped reads. Genes with FDR < 0.05 were considered as significant differentially expressed genes. For heatmaps, log_10_ transformed normalized counts were used.

**Filtering of phase-specific effectors**

For the determination of phase-specific effectors, the following criterium was used: If ${expression}^{Phase X}> \frac{50* {expression}^{Total}}{100}$, where *X* = 1, 2 or 3, is true, then the effector was considered as specific for Phase *X*. As expression, the normalized counts, in counts per million mapped reads, were used. The total expression (*expression^Total^*) was calculated as the sum of the normalized counts in all phases.

**Sensitivity of phase assignment to the classification threshold**

Effectors were assigned to an infection phase when the expression in that phase exceeded a defined fraction of total expression [Phase*ᵢ* / (Axenic + Phase1 + Phase2 + Phase3) > threshold], with a threshold of 0.5 used throughout the main analysis. To assess whether the resulting classification depended on this cut-off, assignments were re-derived for all effectome genes at thresholds of 0.3, 0.4, 0.5, 0.6 and 0.7. For each threshold, the number of effectors assigned to each phase was recorded, and the composition of the Phase 1 set was compared with the 0.5 reference by decomposing it into shared, additionally recruited, and no-longer-assigned genes (Table S7).

**Extraction of promoter sequences**

The 500 or 1000 bp region upstream from the transcription start site was considered as promoter. For the extraction of promoter coordinates for the effector or background genes, the annotation files (GFF3 or GTF) along with the chromosome fasta files were used and the tool *flank* from bedtools was run with the following parameters -l 500 (or 1000 in case of 1000 bp promoter) -r 0 and -s, taking the gene orientation into consideration (Quinlan & Hall, 2010). Thereafter, the generated coordinates were used as input for the extraction of the promoter sequences in fasta format using bedtools *getfasta*.

**Motif enrichment and motif comparison**

The 500 or 1000 bp promoter sequences were used for the motif enrichment analysis with the MEME Suite (Bailey *et al*., 2015). More specifically, the XSTREME tool was used with the following parameters: minimum motif width: 6, minimum motif width: 15, number of motif occurrences per sequence: any. For the motifs from the total effectome promoters, motifs with > 10% enriched sites and *P*-value < 0.05 were considered significant. For the significancy of phase-specific motifs, the thresholds for enriched sites > 55% and *P*-value < 0.05 were considered. Each significant motif was further manually curated to produce a consensus sequence. For the identification of phase-specifically enriched motifs, shuffled sequences were used as background, while for promoter motif enrichment of the total effectome, the secretome promoters of each tested species were selected as background. A further enrichment of each phase-specific motif versus the non-effectome background was estimated by counting the occurrences of the consensus motif in the effectome and non-effectome promoters of all tested species, followed by the calculation of the fold change enrichment.

The comparison between motifs was performed with STAMP using the Pearson correlation coefficient as comparison metric, the ungapped Smith-Waterman as alignment method, iterative refinement as the strategy for multiple alignment and tree construction with UPGMA (Mahony & Benos, 2007).Motif comparison to already existing databases was performed with the tool TomTom from the MEME Suite (Bailey *et al*., 2015). For this, the JASPAR CORE (2022) Fungi database was used. Comparisons with *P*-value < 0.05, and only the ones with good alignment of the core bases after manual observation, were considered good for the determination of the transcription factor groups which could be associated with the motif.

**Promoter windows and TSS definition**

Two promoter windows were used, reflecting the different purposes of the two analyses. *De novo* motif discovery (XSTREME) was performed on the 500 bp immediately upstream of the annotated transcription start site (TSS) of each effector gene. This window focuses discovery on the proximal promoter and limits the inclusion of background sequence, which reduces false-positive motif recovery; it also reflects the compact, gene-dense genome of *U. maydis* (20.5 Mb, 6,902 protein-coding genes; Kämper et al., 2006), in which larger upstream windows increasingly overlap neighbouring coding sequences. For the analysis of positional distribution, motif occurrences were instead counted in a symmetric 1 kb window centred on the TSS (±500 bp). This analysis requires motif frequencies to be assessed both upstream and downstream of the TSS, since enrichment confined to the upstream region, rather than distributed symmetrically, is the distribution expected of a *cis*-regulatory element and distinguishes candidate transcription factor binding sites from background occurrences. TSS coordinates were derived strictly from the genome annotation (GCA_000328475.2), and all windows were extracted using BEDTools (Quinlan & Hall, 2010). No experimental, genome-wide single-nucleotide TSS map is available for *U. maydis*, the TSS were therefore annotation-derived.

**Motif presence around the TSS**

To count the presence of each phase-specific motif around the TSS of the *U. maydis* effectome and non-effectome, the HOMER Suite was used (Heinz *et al*., 2010). More specifically, the tool annotatePeaks.pl was deployed in *tss* mode, taking 1000 bp as a size around the TSS, to cover a 500 bp promoter region, and splitting the region into bins of 50 bp. The motif frequencies were then plotted for both effectome and non-effectome.

**Correlation between number of motifs and gene expression**

In order to correlate the phase-specific gene expression potential of the *U. maydis* effectors with the occurrence of each putative phase-specific motif, at first working with one motif at a time, the effectors were split into groups based on the number of motifs present in their 500bp promoter, ranging between 0 and 6. Then, the average expression (normalized counts) for each phase, 1, 2 or 3, was calculated. Data were, afterwards, plotted generating a pattern of expression potential relative to the copies of the motif in the promoter. Significant differences were estimated by a Student’s t‑test, with *P*-value < 0.05 cut-off.

**Generation of mutants and subsequent infections**

gfp was placed under the control of the pep1, stp1, or cmu1 promoter and integrated into the ip locus of U. maydis. For each construct, a 1,000-bp promoter fragment upstream of the respective effector gene was used to drive gfp expression (WT promoter). Phase-associated *cis*-regulatory motifs identified in Figure 1 were mutated either individually or in combination. For pep1, Phase 1 motifs were mutated in mp_1_, Phase 2 and Phase 3 motifs were mutated jointly in mp_2+3_, and all identified motifs were mutated in mp_ALL_. Corresponding motif mutations of mp_1_ and mp_ALL_ were introduced into the stp1 and cmu1 promoters as indicated. In addition, synthetic promoters containing tandem copies of either the intact or mutated Phase 1 motif were generated to drive gfp expression and integrated into the ip locus of U. maydis. Single‑copy integration of all constructs was verified by Southern blotting and Sanger sequencing of the ip locus. Seven-day-old maize seedlings of the cultivar Golden Bantam were infected with the respective U. maydis strains as described previously (Redkar & Doehlemann, 2016).

**qRT-PCR and quantification of GFP expression**

4-cm sections (1 cm below the infection site) of infected maize leaves were taken at 3 dpi, 6 dpi and 10 dpi. The frozen plant tissue was ground into a fine powder using liquid nitrogen followed by the extraction of total RNA using TRIzol (Thermo Fisher, Waltham, USA) according to the manufacturer’s protocol. Subsequently, a DNase I digest (Thermo Fisher) and cDNA synthesis (Thermo Fisher) was performed using the RevertAid H Minus First Strand cDNA Synthesis Kit, according to the manufacturer protocol. Relative expression of *gfp* was measured using the housekeeping gene *ppi* in a qRT-PCR. qRT-PCR measurements were performed using three independent biological replicates, with two technical replicates per biological sample. Technical replicates were averaged before calculation of relative transcript abundance and statistical analysis. For analysis of reporter activity driven by the native *pep1*, *stp1* and *cmu1* promoters (**Figure 4B**), ΔCt values were calculated for each biological replicate as

*ΔCt = Ct_gfp_ - Ct_endogenous effector_*

where the endogenous effector corresponded to *pep1*, *stp1* or *cmu1*, respectively. Relative reporter expression was calculated as 2^-^*^ΔCt^* and normalized to the mean of the corresponding WT promoter at each time point, which was set to 100% for graphical presentation. Statistical analyses were performed on the untransformed *ΔCt* values rather than on relative expression values. For each promoter, a two-way ANOVA including promoter genotype, time and their interaction was performed. Mutant promoter variants were subsequently compared with the corresponding WT promoter within each time point using Dunnett-adjusted multiple comparisons. Statistical significance was defined as P < 0.05.

For synthetic promoter assays (**Figure 4D**), ΔCt values were calculated as

*ΔCt = Ct_gfp_ – Ct_ppi_*

Relative expression was calculated as 2^-^*^ΔCt^.* P1 and P2 constructs were analyzed separately using two-way ANOVA with motif variant and time as factors, including their interaction. Prespecified comparisons between the intact and mutated motif constructs were performed within each time point, and the resulting P values were adjusted for multiple testing using the Holm method. For graphical presentation, means and standard deviations were calculated in ΔCt space and back-transformed to relative expression values, resulting in geometric means and asymmetric error intervals. All statistical tests were two-sided, and *P < 0.05* was considered statistically significant.

**Confocal microscopy and measurement of GFP intensity**

To visualize GFP expression, the Leica TCS SP8 Confocal Laser Scanning Microscope (Leica, Bensheim, Germany) was employed. GFP was excited at 488 nm and detected within the 490‑540 nm range. The microscopy images were processed using the Leica LAS X.Ink software. For quantification of GFP intensity, ImageJ was used.

**References**

Altenhoff, A. M., Levy, J., Zarowiecki, M., Tomiczek, B., Vesztrocy, A. W., Dalquen, D. A., Müller, S., Telford, M. J., Glover, N. M., Dylus, D., & Dessimoz, C. (2019). OMA standalone: orthology inference among public and custom genomes and transcriptomes. *Genome Research*, *29*(7), 1152–1163. https://doi.org/10.1101/GR.243212.118

Anders, S., Pyl, P. T., & Huber, W. (2015). HTSeq-A Python framework to work with high-throughput sequencing data. *Bioinformatics*, *31*(2), 166–169. https://doi.org/10.1093/bioinformatics/btu638

Bailey, T. L., Johnson, J., Grant, C. E., & Noble, W. S. (2015). The MEME Suite. *Nucleic Acids Research*, *43*(W1), W39–W49. https://doi.org/10.1093/NAR/GKV416

Danecek, P., Bonfield, J. K., Liddle, J., Marshall, J., Ohan, V., Pollard, M. O., Whitwham, A., Keane, T., McCarthy, S. A., & Davies, R. M. (2021). Twelve years of SAMtools and BCFtools. *GigaScience*, *10*(2), 1–4. https://doi.org/10.1093/GIGASCIENCE/GIAB008

Heinz, S., Benner, C., Spann, N., Bertolino, E., Lin, Y. C., Laslo, P., Cheng, J. X., Murre, C., Singh, H., & Glass, C. K. (2010). Simple Combinations of Lineage‑Determining Transcription Factors Prime *cis*-Regulatory Elements Required for Macrophage and B Cell Identities. *Molecular Cell*, *38*(4), 576–589. https://doi.org/10.1016/J.MOLCEL.2010.05.004

Käll, L., Krogh, A., & Sonnhammer, E. L. L. (2004). A Combined Transmembrane Topology and Signal Peptide Prediction Method. *Journal of Molecular Biology*, *338*(5), 1027–1036. https://doi.org/10.1016/J.JMB.2004.03.016

Kämper, J., Kahmann, R., Bölker, M., Ma, L. J., Brefort, T., Saville, B. J., Banuett, F., Kronstad, J. W., Gold, S. E., Müller, O., Perlin, M. H., Wösten, H. A. B., De Vries, R., Ruiz-Herrera, J., Reynaga-Peña, C. G., Snetselaar, K., McCann, M., Pérez-Martín, J., Feldbrügge, M., … Birren, B. W. (2006). Insights from the genome of the biotrophic fungal plant pathogen *Ustilago maydis*. *Nature 2006 444:7115*, *444*(7115), 97–101. https://doi.org/10.1038/nature05248

Kim, D., Paggi, J. M., Park, C., Bennett, C., & Salzberg, S. L. (2019). Graph-based genome alignment and genotyping with HISAT2 and HISAT-genotype. *Nature Biotechnology 2019 37:8*, *37*(8), 907–915. https://doi.org/10.1038/s41587-019-0201-4

Lanver, D., Müller, A. N., Happel, P., Schweizer, G., Haas, F. B., Franitza, M., Pellegrin, C., Reissmann, S., Altmüller, J., Rensing, S. A., & Kahmann, R. (2018). The biotrophic development of *Ustilago maydis* studied by RNA-seq analysis. *Plant Cell*, *30*(2), 300‑323. https://doi.org/10.1105/tpc.17.00764

Mahony, S., & Benos, P. V. (2007). STAMP: a web tool for exploring DNA-binding motif similarities. *Nucleic Acids Research*, *35*(suppl_2), W253–W258. https://doi.org/10.1093/NAR/GKM272

Quinlan, A. R., & Hall, I. M. (2010). BEDTools: a flexible suite of utilities for comparing genomic features. *Bioinformatics*, *26*(6), 841. <https://doi.org/10.1093/BIOINFORMATICS/BTQ033>

Redkar, A. and Doehlemann, G. (2016). *Ustilago maydis* Virulence Assays in Maize. Bio-protocol 6(6): e1760. DOI: 10.21769/BioProtoc.1760.

Robinson, M. D., McCarthy, D. J., & Smyth, G. K. (2009). edgeR: A Bioconductor package for differential expression analysis of digital gene expression data. *Bioinformatics*, *26*(1), 139–140. <https://doi.org/10.1093/bioinformatics/btp616>

Sperschneider, J., & Dodds, P. N. (2022). EffectorP 3.0: Prediction of Apoplastic and Cytoplasmic Effectors in Fungi and Oomycetes. *Molecular Plant-Microbe Interactions*, *35*(2), 146–156. https://doi.org/10.1094/MPMI-08-21-0201-R

Teufel, F., Almagro Armenteros, J. J., Johansen, A. R., Gíslason, M. H., Pihl, S. I., Tsirigos, K. D., Winther, O., Brunak, S., von Heijne, G., & Nielsen, H. (2022). SignalP 6.0 predicts all five types of signal peptides using protein language models. *Nature Biotechnology*, *40*(7), 1023–1025. <https://doi.org/10.1038/s41587-021-01156-3>

Train, C. M., Glover, N. M., Gonnet, G. H., Altenhoff, A. M., & Dessimoz, C. (2017). Orthologous Matrix (OMA) algorithm 2.0: more robust to asymmetric evolutionary rates and more scalable hierarchical orthologous group inference. *Bioinformatics*, *33*(14), i75‑i82. https://doi.org/10.1093/BIOINFORMATICS/BTX229
